# Development of a Bioactive Sauce Based on Oriental Mustard Flour with Antifungal Properties for Pita Bread Shelf Life Improvement

**DOI:** 10.3390/molecules24061019

**Published:** 2019-03-14

**Authors:** Raquel Torrijos, Tiago M. Nazareth, Júlia Pérez, Jordi Mañes, Giuseppe Meca

**Affiliations:** Laboratory of Food Chemistry and Toxicology, Faculty of Pharmacy, University of Valencia, Av. Vicent Andrés Estellés s/n, 46100 Burjassot, Spain; raquel.torrijos@uv.es (R.T.); demena@alumni.uv.es (T.M.N.); jupea2@alumni.uv.es (J.P.); jorge.manes@uv.es (J.M.)

**Keywords:** active packaging, antifungal properties, AITC, bread, shelf life, OTA

## Abstract

Ochratoxin A (OTA) is a mycotoxin produced in the secondary metabolism of fungus belonging to the genus *Aspergillus* and *Penicillium*. In this study, the employment of oriental mustard flour (OMF) as an ingredient in a packaged sauce was evaluated for the generation in situ of the antimicrobial compound allyl isothiocyanate (AITC) in order to preserve pita bread contaminated with *Penicillium verrucosum* VTT D-01847, an OTA producer, in an active packaging system. Four different concentrations (8, 16, 33 and 50 mg/g) were tested. Mycelium formation, mycotoxin production, AITC absorbed by the food matrix, and volatilization kinetics were studied for each concentration. The results obtained were compared with bread treated with the commercial additive calcium propionate (E-282). The results showed a shelf life increase of two and three days with the employment of 33 and 50 mg/g of OMF, with a significant reduction of the fungal population (3.1 and 5.7 logs, respectively) in comparison with the control experiment. The use of 16 and 33 mg/g of OMF in the sauce formulation decreased the concentration of OTA in the bread samples while no OTA production was detected employing 50 mg/g of OMF.

## 1. Introduction

Bread is a staple food consumed around the world and, like other perishable products, is susceptible to fungal contamination. Therefore, the fungal spoilage is a concern on bakery industry, representing a significant source of economic losses and a potential risk to human health due to the production of mycotoxin by toxigenic fungi, mainly from genus *Aspergillus* and *Penicillium* [1]. Bread possess a relatively high water activity (a_w_ 0.94–0.97) with a pH ranging from approximately 7 to 8.6 [2]. These properties are favorable for the germination and growth of a wide range of molds. The loaves of bread have a higher probability of fungal growth since they are commonly sliced. This process increases the surface area for microbial contamination; moreover, the slicing machine can be a vector of spoilage agents [3].

Microbial spoilage reduces the shelf life of food and compromises the safety of consumers with the consequent economic loss for the industry. In this context, synthetic additives are widely used in the food industry and play an important role in the preservation of food quality as well as inhibiting the growth of spoilage and pathogenic microorganisms [4]. Nowadays, consumers demand a reduction of synthetic additives in food due to the concern of the effect of these substances on health. For this reason, the increment in the food of antimicrobial substances from natural sources may be an alternative to increase the shelf life and safety of products, satisfying the consumer requirements [5].

A healthy diet should include vegetables of Brassica genus such as broccoli, Brussels sprouts, cabbage or cauliflower. These vegetables are health-promoting due to the high concentration of ascorbic acid, selenium, soluble fiber and glucosinolates (GTS). Among these compounds, the GTS are secondary metabolites that have been extensively studied in the past decades. Depending on their side chain can be classified in aromatic, indolic or aliphatic compounds [6]. After tissue damage, the GTS is hydrolyzed by enzymes denominated myrosinases. These enzymes are stored separately from GTS in vacuoles inside the plant cell [7]. After the tissue disruption, the myrosinase cleaves the thioglucosides releasing an unstable aglycone named thiohydroximate-*O*-sulphonate. This substance spontaneously rearranges in various products such as isothiocyanates (ITCs), nitriles, organic thiocyanates or epithionitriles depending on the pH of the medium, the presence of specific proteins and particular structural prerequisites [8].

The ITCs are unique in comparison to other essential oils, since they are formed only when the plant cell undergoes some type of injury such as insect bite, grinding, milling or fungi contamination, in addition to the presence of water [9]. Consequently, ITCs are not present in mustard flour, being formed slowly after the addition of water. The antifungal activity of ITCs has been widely studied in the last decade due to its biocidal activity against microorganisms, including bacteria [10], fungi [11], nematodes [12] and insects [13]. The mechanism of action of ITCs are not clearly described but within the cell, ITCs react with nonspecific molecules in the plasm such as saccharides, amino acids, proteins, and lipids, essential components for maintaining life [14].

The main ITC generated by the hydrolysis of the glucosinolate sinigrin give rise of allyl isothiocyanate (AITC), which has shown antimicrobial activity at low concentration against pathogenic microorganisms [15,16].

The employment of AITC as an antimicrobial compound from natural sources has been analyzed in several perishable food matrices like tuna meet [17], pork sausage [18], chopped beef [19], dry-cured ham [20] and cheese [21].

The purpose of this study was to elaborate an active packaging system containing a packaged sauce of oriental mustard flour (OMF) to generate allyl isothiocyanate (AITC) in order to prevent the *Penicillium verrucosum* growth and ochratoxin A production in pita bread. In addition, the kinetics of volatilization and residual concentrations of AITC in the food matrix were evaluated to know the evolution of the antimicrobial compound during the storage period.

## 2. Results and Discussion

### 2.1. Residual Czoncentration of AITC in Plastic Tray Headspace and Pita Bread

**Figure 1:**
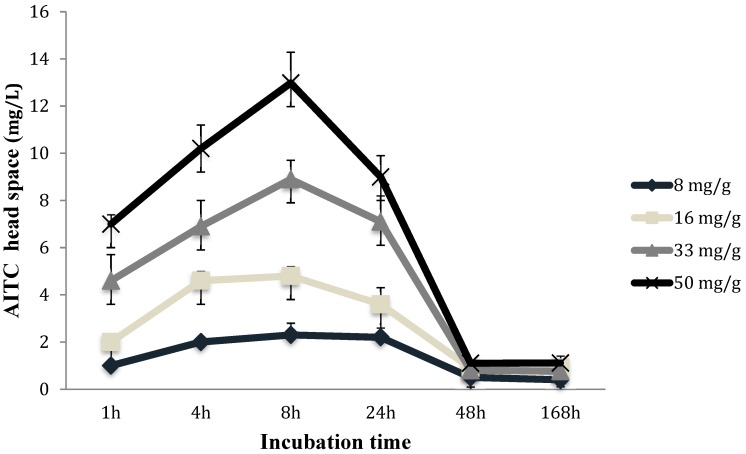
Volatilization kinetics of the AITC contained in the plastic trays produced through the sinigrin conversion obtained from 8, 16, 33 and 50 mg/g of oriental mustard flour incorporated in the bioactive sauce.

The addition of OMF as an ingredient in the sauce allowed the hydrolysis of sinigrin to generate AITC. Consequently, the AITC was volatilized reducing the fungal growth of the *P. verrucosum* in pita bread. The samples were studied using four different OMF concentrations: 8, 16, 33 and 50 mg/g. In particular, the concentration of AITC released by the sauce in the headspace of the plastic tray was proportional to the quantity of mustard flour used in the sauce formulation (Figure 1). After 1h of incubation, the AITC detected in the headspace ranged from 1 to 7 mg/L to the treatments of 8 and 50 mg/g, respectively. These concentrations increased up to 8 h of incubation, which was the point time with a maximum concentration of AITC in the headspace, reaching values of this antifungal compound ranging from 2.3 to 12.9 mg/L for the treatments of 8 and 50 mg/g of OMF, respectively. As showed in Figure 1, after 8 h, the AITC detected in the plastic tray volume decreased up to obtaining constant concentrations at day seven of incubation that ranged from 0.4 to 1.1 mg/L of AITC. It is important to emphasize that the concentrations of AITC detected during all the storage period of the pita bread and considering all the quantity of the oriental mustard flour used for the sauce development are close to the inhibitory concentration referenced by Lopes et al. [22] against *Penicillium nordicum* (0.25, 0.5, 1 and 2 mg/L).

Related with the amount of the AITC absorbed by the pita bread during the storage period of evaluation, the results of this determination are shown in Table 1. In particular, at 7 days of incubation, the concentration of the AITC detected on bread ranged from 4.3 to 59.3 mg/kg depending by the dose of the OMF used for the sauce formulation. Analyzing the amount of this antifungal compound after the storage period stipulated in this study and in particular, after 14 days, it is possible to observe that the AITC was detected just in the bread treated with 33 and 50 mg/g of the OMF with concentrations ranged from 15.9 to 25.6 mg/kg, respectively. These concentrations observed at day 14 were lower in comparison with those detected at day 7, probably due to that the sinigrin present in the OMF could be hydrolyzed in other substances with less antimicrobial activity such as thiocyanates and nitriles [23]. In addition, the AITC generated by hydrolysis of sinigrin can react with group amino of several peptides in the matrix to form a conjugate and reducing its concentration [24].

The EFSA (European Food Safety Authority) described the safety of AITC when used as a food preservative or active packaging [25]. Considering the weight of our product (40 g) and the concentration of AITC absorbed at day 7, the treatments of 33 and 50 mg/g showed a total concentration of 1.93 and 2.37 mg of AITC, respectively. These values were higher than the established for the EFSA for the acceptable daily intake of 0.02 mg/kg BW/day. However, the exposition of AITC by adult consumers from different sources could exceedance from 2 to 8 times the total daily exposure of AITC without toxic effects demonstrated. After day 7, the total concentration of AITC gradually decreased and all treatments showed values above the 0.02 mg/kg BW/day. 

Nielsen and Rios evaluated the sensorial effect of AITC in bread packed [26]. The sensorial test was realized with the untrained panel and the results suggested that the judges did not identify doses above 2 mg of AITC. Therefore, after 7 days of storage, the AITC could be undetectable to the human palate.

### 2.2. Shelf Life Improvement of Pita Bread Contaminated with P. verrucosum and OTA Production

Related with the shelf life results of pita bread contaminated with *P. verrucosum* and treated with the bioactive sauce elaborated with different quantities of OMF the data is showed in Table 2. In particular, in the control group, a visible growth of the *P. verrucosum* was detected at day three of incubation, whereas the commercial control formulated with calcium propionate presented a visible fungal growth at day five with an increase of the bread shelf life in comparison with the control of 2 days. The pita bread treated with the bioactive sauce prepared with 8 mg/g of the OMF presented a shelf life of 4 days, whereas the application of 16 mg/g of OMF in the sauce obtained the same shelf life of the pita bread with commercial control (E-282 additive). The pita bread exposed to the sauce containing 33 mg/g of the OMF presented an increase of the shelf life in comparison with the commercial control and control of 3 and 1 days, respectively. Using the OMF in the quantity of 50 mg/g for the antifungal sauce formulation, any visible *P. verrucosum* growth has been observed on the pita bread during the incubation period analyzed.

**Figure 2:**
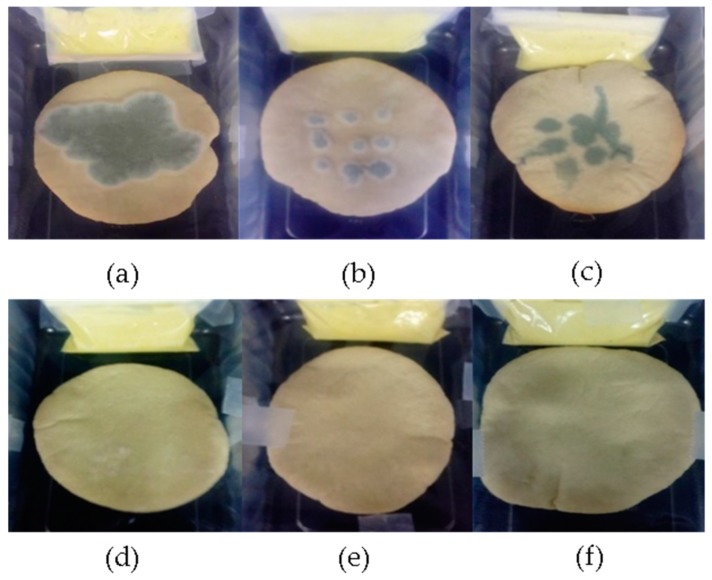
Pita bread inoculated with *P. verrucosum* and stored during 7 days in a plastic tray containing a small sachet sealed with (**a**) control experiment (**b**) pita bread treated with the preservative compound E-282, (**c**) 8 mg/g, (**d**) 16 mg/g, (**e**) 33 mg/g, and (**f**) 50 mg/g of oriental mustard flour sauce.

**Figure 3:**
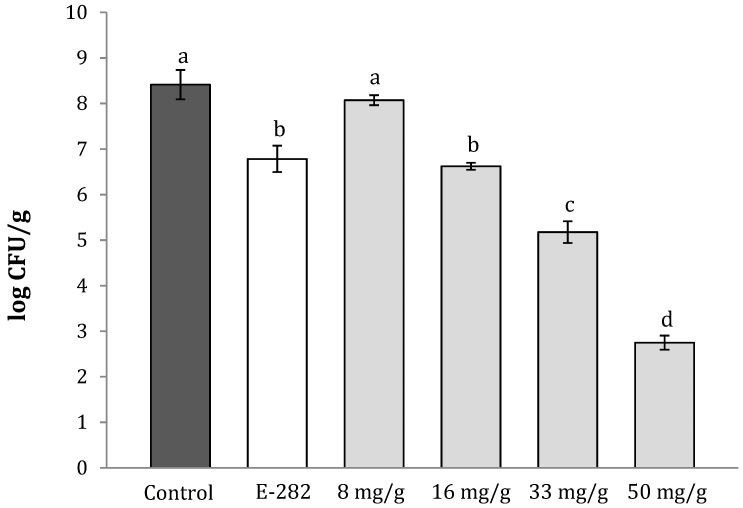
*P. verrucosum* growth in pita breads stored during 7 days in a plastic tray containing a small sachet sealed with 8, 16, 33 and 50 mg/g of oriental mustard flour sauce. Different letters show significant differences among treatments (*p* ≤ 0.05).

The results of visible fungal growth on the pita bread exposed to the AITC vapors generated by sinigrin conversion are according to the microbial population detected in the bread after 7 days of incubation (Figure 2). In particular, as shown in Figure 3, the control experiment presented 8.3 logs UFC/g, whereas the bread produced with the E-282 additive, evidenced fungal contamination of 6.8 logs UFC/g, 1.5 logs lower than the data observed in the control experiment. The application of 8 mg/g of the OMF did not produce any significant decrease in the fungal population when compared to the control experiment and with the bread baked with E-282, whereas using 16, 33 and 50 mg/g of the OMF, the log of the UFC/g of *P. verrucosum* detected was of 6.8, 5.2 and 2.6, respectively. The log reduction of *P. verrucosum* for the treatments of 16, 33 and 50 mg/g was the 1.5, 3.1 and 5.7, respectively, in comparison to the control group.

Clemente et al. analyzed the use of benzyl isothiocyanate (BITC) incorporated in an active packaging against *A. ochraceus.* The use of BITC in vapor phase inhibited the fungal growth by promoting morphological alteration and cellular damage depending on the contact with the antifungal compound [27]. Likewise, the use of AITC reduced the fungal growth of *A. flavus* over time. These results suggested that AITC could also generate to cell damage.

**Figure 4:**
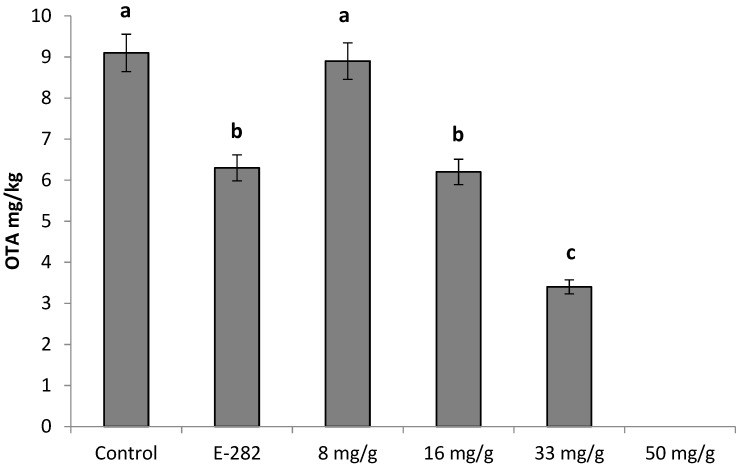
OTA produced by *P. verrucosum* growth in pita breads stored during 7 days in a plastic tray containing a small sachet sealed with 8, 16, 33 and 50 mg/g of oriental mustard flour sauce. Different letters show significant differences between treatments (*p* ≤ 0.05).

Considering that the strain of the *P. verrucosum* used in this study is OTA producer, the data related to the production of this toxic compound in pita bread are plotted in Figure 4. In particular, after 7 days of incubation, the control experiment presented 9.1 mg/kg of the OTA, whereas, on the pita bread baked with the preservative compound E-282, the concentration of this toxic compound detected was 6.3 mg/kg. In the pita bread sealed in the plastic tray and treated with the bioactive sauce with 8 mg/g of the OMF, no statistically differences on the OTA production was detected in comparison to the control group. On the other hand, the treatments of 16 and 33 mg/g of the OMF showed 6.2 and 3.4 mg/kg of OTA in the bread on day 7, respectively. These values are significantly different from the control group, respectively.

All treatments demonstrated values of OTA higher than 3.0 µg/kg stipulated by CE (1881/2006) for processed cereal products [28]. However, with the increment of 50 mg/g of the OMF in the sauce was able to avoid the OTA production to not detected levels, showing an antifungal and anti-mycotoxigenic potential of the OMF as an ingredient in the sauce.

In the study of Quiles et al. [29], active packaging containing AITC or OMF were evaluated as preservatives in pizza crust inoculated with *Aspergillus parasiticus* as aflatoxin producer. The inhibition of *A. parasiticus* growth was detected after 30 days with the employment of gaseous AITC at 5 mL/L and 10 mL/L, sachets containing 5 mL/L and 10 mL/L of AITC and OMF sachet elaborated with 850 mg more 850 mL of water. These authors demonstrated that the application of OMF sachet at 850 mg + 850 mL of water could completely avoid the aflatoxins (AFs) production. These results corroborate with our data since the implementation of OMF in concentrations higher than 50 mg can reduce mycotoxin production by fungal inhibition.

Quiles et al. [30] investigated the production of AFs (B1, B2, G1, G2) by *A. parasiticus* in wheat tortillas treated with yellow and oriental mustard flour incorporated into an active packaging system. The treatment consisted of 0.1, 0.5 and 1 g of yellow or oriental mustard flour mixed with 2 mL of water, with a storage period of 1 month. The AITC generated from the sinigrin hydrolyzed in OMF and the p-hydroxybenzyl isothiocyanate (p-HBITC) obtained from yellow mustard flour inhibited the mycotoxin production. However, the treatments with OMF were more effective. With the employment of 1 g of OMF showed reductions of 90% in AFs B1, B2, G1, and G2 production, while p-HBIYC demonstrated an average of 17.7 to 45.2% on mycotoxin reduction.

Saladino et al. [31] evaluated the capacity antimicotoxigenic of OMF employed in piadina at concentrations ranging from 0.1 to 1g in active packaging. The results demonstrated that OMF reduced the *A. parasiticus* growth by 12.2% to 80.6%. In addition, the ITC generated in active packaging avoided the AFs synthesis by 60.5% to 89.3%, depending on the quantity of the mustard flour used.

Saladino et al. [3] studied the mycotoxin reduction and the fungal growth inhibition of *A. parasiticus* in loaf bread by the employment of allyl, benzyl and phenyl isothiocyanates. Treatments consisted in paper filters or small plastic bags paper filters soaked with AITC, BITC or PITC at three different concentrations (0.5, 1 and 5 mL/L) and introduced into a plastic tray with the loaf bread. The study evidenced an increase of the shelf life with the employment of 5 mL/L of AITC. In addition, this treatment showed the highest reduction of the AFs content (above 60%).

## 3. Materials and Methods

### 3.1. Chemicals and Microorganisms

OTA (purity >99%), formic acid (analytical grade, purity >98%), ammonium formate (analytical grade, purity ≥99.0%) and calcium propionate (E-282) were purchased from Sigma Aldrich (St. Louis, MO, USA). Methanol (LC-MS grade, purity ≥99.9%) was obtained from Fisher Scientific (Hudson, NH, USA). Deionized water (<18 MΩ cm resistivity) was acquired from a Milli-Q Millipore water purification system (Massachusetts, United States). Culture media potato dextrose for agar (PDA), potato dextrose for broth (PDB) and buffered peptone water were purchased from Liofilchem Bacteriology Products (Roseto, Italy). The strain of *Penicillium verrucosum* VTT D-01847 was obtained from the VTT Technical Research of Finland LTD (VTT, Otaniemi, Finland). The microorganism was maintained in sterile glycerol at −80 °C. Then, it was recovered in PDB broth at 25 °C for 48 h prior to use.

### 3.2. Samples Preparation and Antifungal Treatment

The pita bread recipe included 250 g of wheat flour, 2.5 g of sugar, 5 g of NaCl, 15 g of yeast bakery products (Levital, Spain), 10 mL of olive oil and 125 mL of warm water. Briefly, all ingredients were placed in a recipient mixed and kneaded manually for 15 min. Then, the dough produced was fermented for 40 min at room temperature. Posteriorly, the dough was divided into 9 portions of 40 g, flattened and baked at 180 °C for 5 min using a deck oven (MIWE, Arnstein, Germany). Each pita bread was inoculated with 100 µL of a 0.1% buffered peptone water suspension containing 4 × 10^5^ conidia/mL of *P. verrucosum* (VTT D-01847) in 9 points equidistant. The control group did not receive treatment and, the commercial control was prepared with 2 g/kg of calcium propionate (E-282), which is a common preservative granted as an ingredient in bakery products [32].

The conidial suspension was previously determined as described by Kelly et al. [33]. The conidia were harvested from the potato dextrose agar plates with sterile water and scraping the colonies. Then, the spore concentration was measured by optical density at 600 nm and adjusted in a buffered peptone water medium.

The sauce was elaborated with 125 g of yogurt, 10 mL of mustard sauce and 10 mL of honey. In addition, the antifungal sauce was produced adding OMF at the doses of 8, 16, 33 and 50 mg/g to the basic ingredients. To improve the conversion of the glucosinolate sinigrin into AITC, OMF was previously mixed with 2 mL of distilled water and then added to the sauce formulation. Each plastic bag contained 30 g of sauce and was closed hermetically.

A commercial control and a control group were used for each set of assays, being packaged with sauce without OMF incorporated in the formulation. The sauces were packaged with bread in polyethylene trays, closed hermetically and incubated during 7 days at room temperature. During the storage period, the visible fungal growth was evaluated to establish the effect of treatments in the shelf life of the pita breads. After the incubation period, the sampling was carried out to determine the fungal population and to quantify the OTA production. All analyses were carried out in triplicate (n = 9).

### 3.3. Ochratoxin A Extraction

OTA extraction was carried out using the method described by Saladino et al. [3] with some modification. The samples of pita bread (40 g) were finely crushed in a grinder (Oster, Valencia, Spain). Posteriorly, 5g of the sample was weighed in Falcon tubes (50 mL) containing 25 mL of methanol and homogenized employing an Ultra Ika T18 basic Ultraturraz (Staufen, Germany) at 10000 rpm for 3 min. Then, the extracts were centrifuged at 4 °C with a rotation of 4000 rpm for 5 min, and the supernatant was recovered and evaporated using a Büchi Rotavapor R-200 (Postfach, Switzerland). The residue obtained was resuspended in 5 mL of methanol, transferred to a 15 mL falcon tube and evaporated with gaseous nitrogen at 35 °C in a Turbovap Evaporator (Zymark, Hopkinton, MA, USA). The dry extract obtained was recovered with 1 mL of methanol (100%) filtered through a 0.22 mM syringe filter, transferred to a glass vial and injected into an LC-MS/MS system.

### 3.4. Ochratoxin A Identification and Quantification by LC-MS/MS

OTA analyses were performed using a liquid chromatograph system Agilent 1200 chromatograph (Agilent Technologies, Palo Alto, CA, USA) associated a mass spectrometer 3200QTRAP (AB Sciex, Foster City, CA, USA) and equipped to an interface of electrospray ionization (ESI). The software used to process the data was Analyst version 1.5.2 for the Windows. The stationary phase was an analytical column of reversed phase (Gemini C18 column, 150mm × 2mm × 3 mm) (Phenomenex, Madrid, Spain). The mobile phases employed as eluents consisted of water 0.1% formic acid with 5 mM ammonium formate (Sigma-Aldrich, St. Louis, MI, USA) (A) and methanol with 5 mM ammonium formate and formic acid at 0.1% (B). Elution gradient was established as follows. The initial condition was 10% B increasing to 80% in 1.5 min and kept up to 2.5 min. Afterward, eluent B was increased to 90% in 6 min and later 100% was obtained in 4 min. Posteriorly, the initial conditions (B: 10%) were reestablished for 5 min. The flow rate was set at 0.25 mL/min in all steps. Injection volume was 20 µL. MS/MS analysis was obtained applying the parameters: the ion spray voltage at 5500 V; source temperature, 450 °C; curtain gas, 20 psi; the ion source gas 1 to sheath gas at 50 psi; the ion source gas 2 or drying gas at 55 psi. The gas used to nebulize and the collision was nitrogen. The precursor-to-product ion transition was *m/z* 404.3/102.1-404.3/239.0 and 404.3/358.1 for OTA.

A calibration curve was previously prepared with standards of OTA at concentrations ranging from 0.1 to 100 µg/L. The peak areas obtained in the standard curve was compared to the values found in the samples to quantify the OTA production.

### 3.5. Shelf Life and Determination of the Fungal Population

The breads contained in the polyethylene trays were examined daily to determine its shelf life. When breads showed a visible sign of fungal growth, the shelf life was closed, considering that consumers would eventually reject the product [34].

The determination of the fungal population was performed after 7 days of incubation. The breads (40 g) were transferred to sterile plastic bags containing 160 mL of sterile peptone water (0.1%) and shaken with a stomacher (IUL, Barcelona, Spain) for 40 s. The mixture obtained was serially diluted in sterile 15 mL plastic tubes and then, aliquots of 100 μL of each dilution were placed in PDA plates and incubated at 25 °C for 3 days.

### 3.6. Extraction and Analysis of AITC in Pita Bread

The AITC extraction and quantification from pita bread were carried out as described by Nazareth et al. [35] with some modifications. 

Each pita bread (40 g) was added to hermetic tubes of 150 mL containing 80 mL of methanol. The mixture was extracted for 30 min in a water bath at 40 °C and 10 min in an ultrasonic bath. Then, the extract was centrifuged at 4000× g for 5 min at 20 °C. The supernatant was recovered and filtered through a nylon membrane filter (0.22 μm) and an aliquot of 10 μL was injected in a gas chromatograph.

The residual AITC absorbed by samples was quantified using a gas chromatograph (GC) coupled a flame ionization detector (FID) (GC 6890, Agilent Technologies Inc., Santa Clara, CA, USA), equipped with a fused capillary column (CP-SIL 0.25mm × 30m) (Varian, Middelburg, Netherlands). The inlet temperature was set at 200 °C with 250 °C of detector temperature. H_2_ at 5 ml/min was the carrier gas, and the FID gasses were H_2_ at 40 mL/min, and purified air at 450 mL/min. The temperature program was a gradient when the initial temperature was 60 °C for 1 min, increased at 8 °C/min up to 100 °C and held for 5 min, then the temperature was raised at 15 °C/min up to 200 °C, the time of analysis was 16.6 min per sample. 

Identification and quantification of AITC were carried out comparing the samples areas with points standards curve (1–100 mg/L).

### 3.7. Headspace Analysis of AITC

The AITC evaporated in the headspace of the plastic trays was determined during the storage period (7 days), injecting 100 mL of the headspace through a septum applied in the tray cover in a GC system. GC parameters employed for the identification and quantification of AITC were identical as described for the determination of AITC in the food matrix in paragraph 3.6. Three replicates were carried out for each test condition.

### 3.8. Statistical Analysis

GraphPad Prism version 6.0 (GraphPad Software Inc., La Jolla, CA, USA) was used for the statistical analysis of data. Differences among groups were realized using analysis of variance ANOVA followed by Tukey’s multiple comparison tests. Statistical differences were considered significant if *p* ≤ 0.05.

## 4. Conclusions

In summary, this paper showed the capacity of AITC released from a packaged bioactive sauce elaborated with OMF to reduce the growth of the toxigenic fungi *P. verrucosum* in pita bread. The results demonstrated that the employment of this ingredient reduces the production of OTA by the fungi with a dose depending effect. In conclusion, OMF could be potentially used as a precursor of antimicrobial substances against *Penicillium* species, commonly found as contaminants in bakery products. Considering the current trend of reducing the presence of synthetic chemical additives in food, the employment of natural ingredients that releases antimicrobial substances such as OMF may be an alternative to food preservation rather than traditional additives.

## Figures and Tables

**Table 1:** AITC adsorbed (mg/kg) by the pita bread, treated with different concentration of oriental mustard flour sauce contained in a bioactive sachet and introduced inside the bread packaging.

Treatment	Day		
	0	7	14
Control	Nd	nd	nd
8 mg/g	Nd	4.3 ± 1.9a	nd
16 mg/g	Nd	9.5 ± 0.2a	nd
33 mg/g	Nd	48.3 ± 6.0b	15.9 ± 3.0a
50 mg/g	Nd	59.3 ± 15.1b	25.6 ± 3.1b

Different letters show significant differences among treatments (*p* ≤ 0.05).

**Table 2:** The shelf life of pita bread, stored in a plastic tray, contaminated with *P. verrucosum* and treated with 8, 16, 33 and 50 mg/g of oriental mustard flour sauce, in comparison to the control experiment and the pita bread treated with the preservative compound E-282.

Treatment	Days				
	3	4	5	6	7
Control	+	+	+	+	+
E-282	−	−	+	+	+
8 mg/g	−	+	+	+	+
16 mg/g	−	−	+	+	+
33 mg/g	−	−	−	+	+
50 mg/g	−	−	−	−	−

(+) Positive growth; (−) Negative growth.
